# SNX8 Enhances Non-amyloidogenic APP Trafficking and Attenuates Aβ Accumulation and Memory Deficits in an AD Mouse

**DOI:** 10.3389/fncel.2019.00410

**Published:** 2019-09-06

**Authors:** Yongzhuang Xie, Mengxi Niu, Chengxiang Ji, Timothy Y. Huang, Cuilin Zhang, Ye Tian, Zhun Shi, Chen Wang, Yingjun Zhao, Hong Luo, Dan Can, Huaxi Xu, Yun-wu Zhang, Xian Zhang

**Affiliations:** ^1^Fujian Provincial Key Laboratory of Neurodegenerative Disease and Aging Research, Institute of Neuroscience, School of Pharmaceutical Sciences, School of Medicine, Xiamen University, Xiamen, China; ^2^Neuroscience Initiative, Sanford Burnham Prebys Medical Discovery Institute, La Jolla, CA, United States; ^3^The United Innovation of Mengchao Hepatobiliary Technology Key Laboratory of Fujian Province, Mengchao Hepatobiliary Hospital of Fujian Medical University, Fuzhou, China; ^4^Department of Neurology, The First Affiliated Hospital, School of Medicine, Xiamen University, Xiamen, China

**Keywords:** SNX8, sorting nexins, Alzheimer’s disease, β-amyloid, β-amyloid precursor protein, APP trafficking, sAPPα

## Abstract

Dysregulation of various APP trafficking components in the endosome has been previously implicated in Alzheimer’s disease (AD). Although single nucleotide polymorphisms within the gene locus encoding the endosomal component, SNX8 have been previously associated with AD, how SNX8 levels are altered and its contribution to AD onset is currently unknown. Here, we observe decreased expression of SNX8 in human AD and AD mouse brain. SNX8 predominantly localized to early and late endosomes, where SNX8 overexpression enhanced total APP levels, cell surface APP distribution and consequent soluble APPα cleavage. SNX8 depletion resulted in elevated β-amyloid (Aβ) levels, while SNX8 overexpression reduced Aβ levels in cells and in an APP/PS1 AD mouse model. Importantly, SNX8 overexpression rescued cognitive impairment in APP/PS1 mice. Together, these results implicate a neuroprotective role for SNX8 in enhancing non-amyloidogenic APP trafficking and processing pathways. Given that endosomal dysfunction is an early event in AD, restoration of dysfunctional endosomal components such as SNX8 may be beneficial in future therapeutic strategies.

## Introduction

Senile plaques and neurofibrillary tangles are two major pathological hallmarks that appear in Alzheimer’s disease (AD) brain. Senile plaques are extracellular deposits comprising β-amyloid (Aβ) peptides proteolytically derived from amyloidogenic cleavage of the β-amyloid precursor protein (APP) (Glenner and Wong, 1984). Neurofibrillary tangles are intracellular paired helical filaments comprising hyperphosphorylated forms of the microtubule-associated protein tau (Buee et al., 2000). Multiple lines of evidence suggest that AD pathogenesis is triggered by the overproduction/excessive accumulation of Aβ, which triggers a cascade of neurodegenerative steps to form senile plaques and intra-neuronal fibrillary tangles, associated with neuronal loss in susceptible brain regions (Hardy and Higgins, 1992).

The neurotoxic Aβ peptide is proteolytically derived from APP by sequential cleavage by β-secretase (BACE1) and γ-secretase enzymes, where β-secretase cleavage is a rate-limiting step in Aβ generation. APP is a type-I transmembrane protein which is processed through amyloidogenic and non-amyloidogenic pathways by intracellular sorting factors. APP is predominantly routed through a constitutive, non-amyloidogenic pathway where newly synthesized APP traffics from the endoplasmic reticulum (ER) through the Golgi/trans-Golgi network (TGN) to the plasma membrane (Xu et al., 1997; Greenfield et al., 1999), whereby APP is cleaved by α-secretases to generate a neuroprotective soluble fragment, sAPPα (Furukawa et al., 1996; Mattson, 1997; Han et al., 2005). Uncleaved APP can be internalized from the cell surface into endosomes; subsequent sorting of APP to acidified late endosomes results in amyloidogenic cleavage, whereas retrograde APP trafficking from the endosome to the Golgi by-passes amyloidogenic cleavage (Nordstedt et al., 1993; Caporaso et al., 1994).

Extensive experimental evidence suggests that internalized APP is routed into amyloidogenic processing pathways, thereby enhancing Aβ generation (Haass et al., 1993a, b). Therefore, APP trafficking from the endosome is critical in determining its proteolytic commitment to amyloidogenic or non-amyloidogenic pathways (Zhang and Xu, 2007; Cao et al., 2018). APP trafficking components that re-route APP from endosomes to the Golgi or cell surface can promote non-amyloidogenic APP processing (Haass et al., 2012). Although this suggests that endosomal trafficking factors may be perturbed in AD, few endosomal APP trafficking components and their modulation in human AD have been described thus far. Interestingly, an AD risk component identified through microarray analysis and subsequent GWAS studies, SORLA (Lambert et al., 2013) was previously shown to bind and traffic APP, and promote its distribution to non-amyloidogenic subcellular compartments such as the Golgi (Andersen et al., 2005) and cell surface (Huang et al., 2016). Although SORLA-mediated retrograde APP trafficking from the endosome to the Golgi may occur through SORLA interactions with the retromer subunit, Vps26 (Dumanis et al., 2015), SORLA may also mediate endosomal APP recycling to the cell surface through interactions with SNX27 (Huang et al., 2016). Significantly, expression of components comprising the core retromer complex such as Vps35 and Vps26 is perturbed in vulnerable brain regions in AD (Small et al., 2005). This suggests that perturbation of amyloidogenic APP trafficking mechanisms from the endosome may be a potential pathogenic mechanism in AD onset.

Sorting nexins (SNXs) are a diverse group of cellular trafficking proteins that characteristically comprise a canonical PX PtdIns(3)P-binding domain that facilitates their localization to PI(3)P in early endosomes. The ability of SNXs to bind specific phospholipids, as well as their propensity to form protein-protein complexes implicate a role for SNXs in membrane trafficking and protein sorting (Cheever et al., 2001; Ellson et al., 2001). Although 33 mammalian SNXs and 10 yeast SNXs have been identified so far, specific functions for many SNXs remain elusive (Cullen, 2008; Zhang et al., 2018). Recently, several SNX members have been found to participate in APP metabolism/Aβ generation through trafficking of various AD-associated proteins. In addition to SNX27 that can interact with SORLA to mediate endosomal APP recycling to the cell surface (Huang et al., 2016), the PX-FERM component SNX17 has been found to interact with APP in early endosomes, where SNX17 downregulation leads to a reduction in steady-state APP levels accompanied by a concomitant increase in Aβ production (Lee et al., 2008). Furthermore, SNX33 (an SH3-PX-BAR containing SNX member) can bind to the endocytic GTPase dynamin; SNX33 overexpression reduces the rate of APP endocytosis in a dynamin-dependent manner, thereby enhancing APP α-secretase cleavage at the cell surface (Schobel et al., 2008). The PX-BAR component SNX6 interacts with BACE1 and impedes retrograde transport from early endosomes to TGN, thereby enhancing BACE1 retention in endosomes (Okada et al., 2010). We recently also identify interactions between BACE1 and SNX12 (comprising a single PX domain) and demonstrate a role for SNX12 in modulating BACE1 distribution to the cell surface and early endosomes (Zhao et al., 2012). Although studies so far suggest that SNXs may have a role in AD, how other PX-BAR SNXs can contribute to AD pathogenesis remains unclear.

The PX-BAR component, SNX8 localizes to early endosomes and facilitates Shiga toxin and ricin trafficking from the endosome to the Golgi network (Dyve et al., 2009). However, an endogenous cargo substrate for SNX8 has not yet been identified. Interestingly, two single nucleotide polymorphisms (rs2286206, a non-coding exon variant and rs10249052, an intronic variant) within the human *SNX8* gene locus are associated with late onset AD (Rosenthal et al., 2012). Herein, we characterized the potential role of SNX8 in AD pathogenesis using cell and animal models. We found that SNX8 levels were dramatically decreased in AD patients and APP/PS1 AD mouse brain. In addition, SNX8 overexpression increased APP protein levels, APP cell surface distribution and sAPPα secretion, and attenuated Aβ levels. Conversely, SNX8 downregulation decreased sAPPα levels and increased Aβ levels. Interestingly, AAV-mediated SNX8 overexpression in APP/PS1 mouse brain reduced Aβ levels and reversed cognitive impairments in Y-maze tests. Together, these results implicate a neuroprotective role for SNX8 in enhancing non-amyloidogenic APP trafficking, thereby suppressing Aβ accumulation and consequent cognitive impairment in AD.

## Materials and Methods

### AD Human Samples

Brain cortical samples from 5 AD patients (age range 76–90 years, Braak stage VI) and 5 controls (age range 71–97 years) were kindly provided by Dr. Eliezer Masliah. Samples were lysed in RIPA lysis buffer and equal protein quantities were subjected to Western blotting to detect SNX8 levels.

### Animals and Tissue Collection

Animals used in this study include male C57BL/6 wild-type mice and APP/PS1 (APPswe/PSEN1dE9) AD models coexpressing the Swedish mutant APP and the exon-9 deletion mutant PS1, provided by Nanjing Biomedical Research Institute of Nanjing University, China. All animal procedures were performed in accordance with the National Institute of Health Guidelines for the Care and Use of Laboratory Animals and were approved by the Laboratory Animal Management and Ethics Committee of Xiamen University.

To collect hippocampal and cortical tissues, mice were anesthetized and transcardially perfused with ice-cold 1 × PBS. After dissecting the brain, hippocampal and cortical tissues were separated, homogenized, and lysed in RIPA lysis buffer (25 mM Tris–HCl, pH 7.6, 150 mM NaCl, 1% sodium deoxycholate, 1% Nonidet P-40, 0.1% sodium dodecyl sulfate) supplemented with the Complete Protease Inhibitor Cocktail (Roche) for 40 min. After centrifugation (12,000 rpm, 30 min), the supernatants were kept at −80°C for further analysis.

### Antibodies

The SNX8 antibody was purchased from Novus. The Aβ (6E10) antibody was purchased from Biolegend. GAPDH, GFAP, and β-actin antibodies were purchased from Cell Signaling Technology. NeuN and Giantin antibodies were purchased from Abcam. The Myc (9E10) antibody was purchased from Santa Cruz Biotechnology. The Iba1 antibody was purchased from Wako. The tau PHF1 antibody, Alexa Fluor 594 goat anti-rabbit IgG, Alexa Fluor 488 goat anti-mouse IgG, Alexa Fluor 635 goat anti-mouse IgG, goat anti-rabbit IgG (H + L) secondary antibody HRP, and goat anti-mouse IgG (H + L) secondary antibody HRP were purchased from Thermo Fisher Scientific. Antibodies against APP (369) and PS1-NTF (Ab14) were generated in-house (Thinakaran et al., 1996; Xu et al., 1997). The sAPPα (B436) antibody has been described previously (Eggert et al., 2009).

### Cells Cultures

Human HEK293T, HEK-swAPP, SH-SY5Y, and Hela cells were maintained in high glucose DMEM (Hyclone) supplemented with 10% fetal bovine serum (FBS, Gibco), 100 units/ml penicillin (Gibco), and 100 μg/ml streptomycin (Gibco).

Primary neurons were isolated from wild-type mice at postnatal day 0 and cultured as previously described (Bobela et al., 2017). Primary microglia and astrocytes were isolated from wild-type mice at postnatal day 1–2 and cultured as previously described (Zeng et al., 2007; Zhong et al., 2018; Zhong et al., 2019).

### DNA Constructs

Myc-tagged SNX8 or APP was cloned using the pCDNA3.1-myc/His (Invitrogen) construct as a backbone; mCherry-tagged SNX8 was cloned using the mCherry-C1 (Clontech) construct as a backbone. GFP-tagged Rab5 plasmid was kindly provided by Dr. Steve Caplan (University of Nebraska, Lincoln, NE, United States); Rab4 (Addgene plasmid #49434) and Rab7 (Addgene plasmid #12605) were kindly provided by Dr. Richard Pagano (Mayo Clinic, Rochester, MN, United States) and obtained from Addgene.

### RNA Interference

shRNA targeting human SNX8 and scrambled shRNA hairpin sequences were cloned into the pLL3.7 vector (a gift from Luk Parijs, Addgene #11795). Targeting sequences within the shRNAs hairpins were as follows: scrambled control 5′-GCCATATGTTCGAGACTCT-3′; SNX8 shRNA: 5′-GATCTTCTCATATTCGGGA-3′.

### Transfection

Human HEK293T, HEK-swAPP, SH-SY5Y or Hela cells were transfected with indicated plasmids using Turbofect transfection reagent (Thermo Fisher Scientific), following the manufacturer’s protocol. For overexpression of proteins, cells were transfected with overexpression plasmids for 24 h; for shRNA expression, cells were transfected with shRNA-expressing plasmids for 48 h.

### Western Blotting

Cells were lysed in TNEN lysis buffer (150 mM NaCl, 50 mM Tris–HCl, pH 8.0, 2 mM EDTA, 1% Nonidet P-40) supplemented with the Complete Protease Inhibitor Cocktail (Roche). Equal amounts of protein lysates from cell lysates, human cortical tissues lysed in RIPA buffer or mouse hippocampal and cortical tissues lysed in RIPA buffer were resolved using SDS-polyacrylamide gel electrophoresis, transferred to polyvinylidene difluoride (PVDF) membranes (Merck Millipore), and probed with primary antibodies as indicated, and detected by chemiluminescence using horseradish peroxidase conjugated secondary antibodies. Protein band intensity was quantified by densitometry by using Image J.

### Quantitative Real-Time PCR

Total RNA was extracted using TRIzol Reagent (Invitrogen), and reverse transcription was performed using the ReverTra Ace qPCR RT Kit (Toyobo), following the manufacturer’s instructions. Equal amounts of cDNA from each sample were subjected to real-time PCR experiments. Primers used in this study were as follows:

SNX8:
  forward primer: 5′-ACTTCTCCCTCTACTGCCTG-3′;
  reverse primer: 5′-GTCATTCCACACCTTGCTCA-3′;
APP:
  forward primer: 5′-GCTGGCTGAACCCCAGATT-3′;
  reverse primer: 5′-CCCACTTCCCATTCTGGACAT-3′;
β-actin:
  forward primer: 5′-ATCAAGATCATTGCTCCTCCT GAG-3′;
  reverse primer: 5′-CTGCTTGCTGATCCACATCTG-3′.

### Cell Surface Biotinylation

Cell surface biotinylation assays were performed as described previously (Mammen et al., 1997; Zhang et al., 2005). Briefly, cells were labeled with EZ-Link^TM^ Sulfo-NHS-SS-Biotin (Thermo Fisher Scientific) at 4°C. Biotinylated proteins in cell lysates were affinity precipiated by Streptavidin Agarose Resin (Thermo Fisher Scientific) and detected by Western blotting.

### Aβ ELISA Assay

Aβ_40_ and Aβ_42_ in conditioned media was quantified by ELISA using Human Aβ_1__–__40__/__1__–__42_ ELISA Kits (Thermo Fisher Scientific), following the manufacturer’s instructions.

### Immunofluorescence

Cells grown on coverslips were fixed in 4% paraformaldehyde, permeabilized with 0.2% Triton X-100 in PBS, incubated with indicated primary and fluorescence-conjugated secondary antibodies, and imaged with a laser scanning fluorescence confocal microscope (Nikon, Japan) using a 100 × oil immersion objective.

### Stereotactic Injection of AAV

AAV8 particles comprising EGFP-SNX8 or EGFP (1.5 μl, titer 2 × 10^12^) expression sequences were stereotactically injected into the two lateral ventricles of wild-type C57BL/6 or APP/PS1 postnatal day 0 (P0) neonates. Ten months after injection, mice were subjected to behavioral testing and subsequently sacrificed for biochemical analyses.

### Behavioral Tests

Experimental mice injected with AAVs were subjected to Y-maze tests to evaluate spontaneous alternation behavior. One mouse was placed in the center of a maze with three equally spaced arms [30 cm (L) × 6 cm (W) × 15 cm (H)] forming a “Y” shape. Total travel distance, time spent in each arm, sequence of arm entries and total number of arms entered from each mouse was recorded using Smart 3.0 video tracking system (Panlab). Spontaneous alternation percentage was defined as the ratio of consecutive specific arm entries to the total entry events observed.

### Statistical Analyses

All statistical analyses were determined using GraphPad Prism 6. Results depicted represent mean ± standard error of the mean (SEM). Statistical significance was assessed by Mann–Whitney *U* test or two-way ANOVA.

## Results

### SNX8 Levels Are Reduced in Human AD and AD Mouse Brain

**FIGURE 1:**
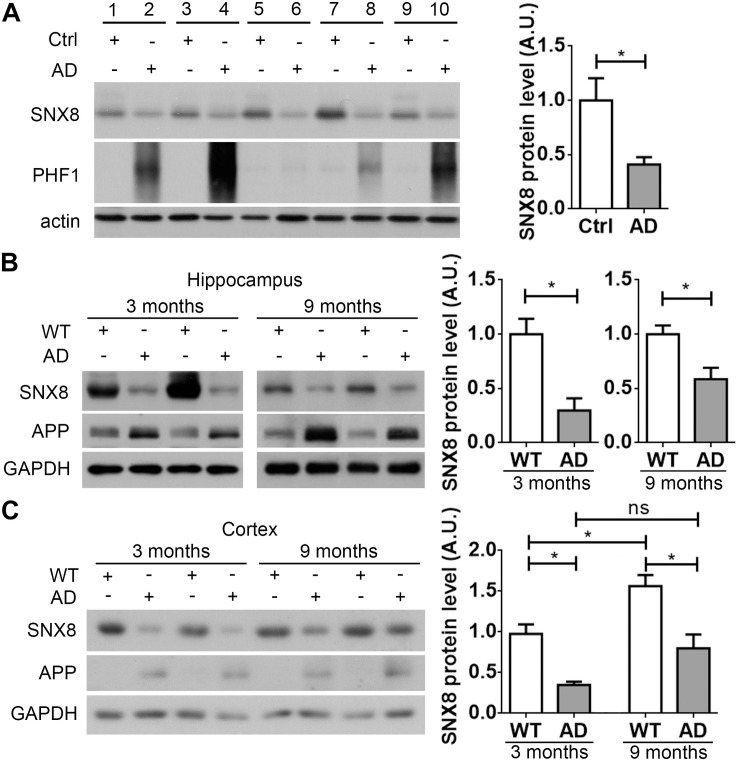
SNX8 levels are reduced in human AD and APP/PS1 AD mouse brain. **(A)** Equal protein quantities in lysates from paired AD patient brains (AD) and age- and sex-matched non-dementia controls (Ctrl) were subjected to Western blotting with antibodies to detect SNX8, phosphorylated tau (PHF1) and β-actin. SNX8 protein intensities were quantified by densitometry, and normalized to respective controls (set to 1.0), *n* = 5, ^∗^*p* < 0.05, Mann–Whitney *U* test. **(B,C)** Equal protein quantities of lysates from hippocampal **(B)** or cortical **(C)** tissues of APP/PS1 AD mice and wild type (WT) littermates at 3 and 9 months of age were subjected to Western blotting with antibodies against SNX8, APP (the 369 antibody recognizing both mouse and human APP was used in **B**; the 6E10 antibody recognizing human APP only was used in **C**) and GAPDH. SNX8 protein intensities were quantified by densitometry, and normalized to respective controls (set to 1.0), *n* = 4, ^∗^*p* < 0.05, Mann–Whitney *U* test.

Given that SNX8 SNPs have been previously identified in AD, SNX8 levels may be altered with AD onset. To test this, we characterized SNX8 levels in human post-mortem brain tissue from AD patients and age/gender matched controls. Our results indicate that SNX8 levels were dramatically reduced in the cortex of AD patients compared to controls (Figure 1A). In addition, we found that SNX8 protein levels were also markedly reduced in the hippocampus (Figure 1B) and the cortex (Figure 1C) of APP/PS1 mice compared to wild type littermates at 3 and 9 months of age. Together, these results indicate that SNX8 levels are attenuated in human AD, and in APP/PS1 AD mouse brain. Interestingly, we noticed that SNX8 levels were significantly increased at 9 months of age compared to those at 3 months of age in WT mice (Figure 1C). While there was a trend of increase in SNX8 levels in 9-month-old AD mice compared to those of 3-month-old AD mice, the increase was not significant (Figure 1C). It is possible that the increase of SNX8 levels during aging is a physiological response, which can be interfered during AD; and this deserves further scrutiny. One thing needs to be clarified here is that elevated APP levels found in APP/PS1 mice (Figure 1B) were detected by using the 369 antibody recognizing both mouse and human APP; and this should reflect overexpression of the human APP transgene *per se* (Figure 1C), and cannot be attributed to the reduction in SNX8 levels, as we demonstrated that downregulation of SNX8 actually reduce APP levels (see below).

### Characterizing SNX8 Expression

**FIGURE 2:**
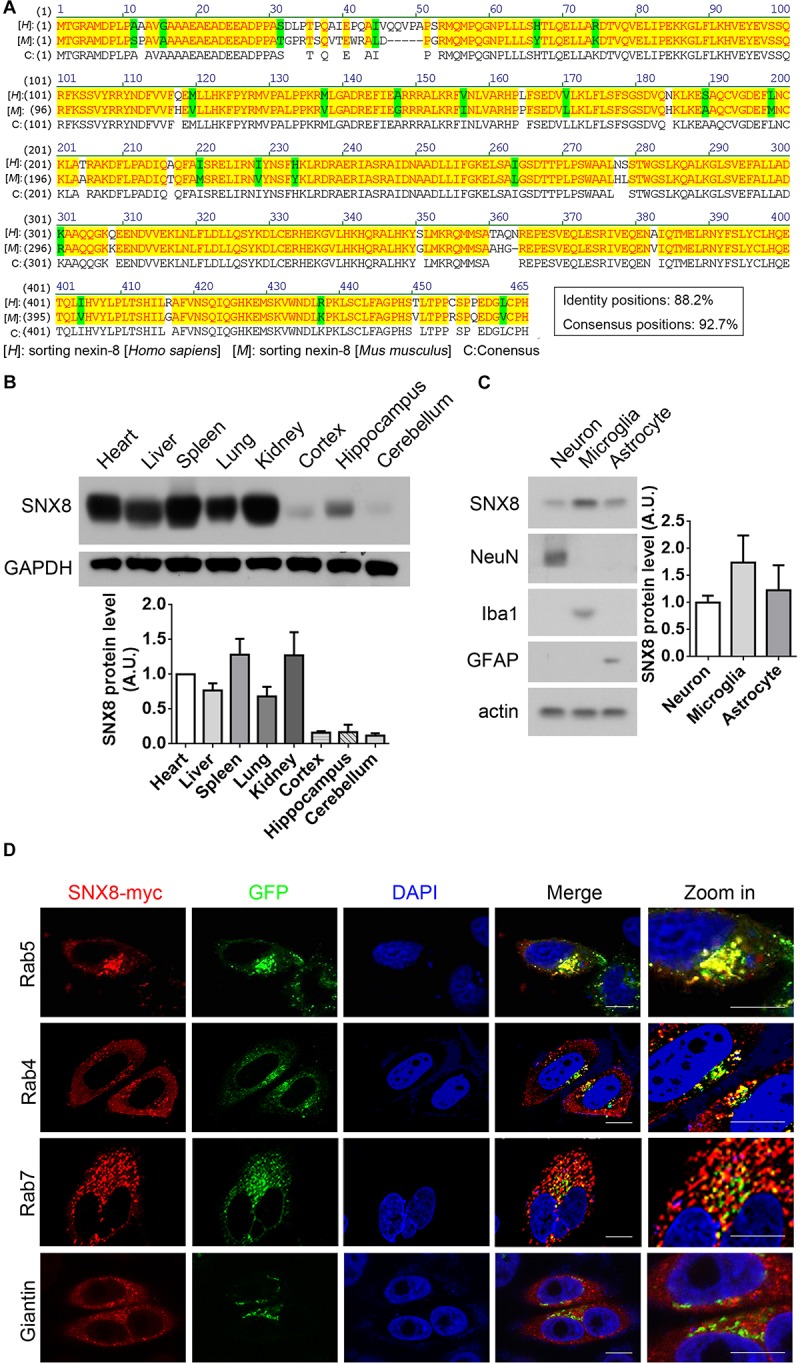
Characterizing SNX8 Expression and Localization. **(A)** Human and mouse SNX8 protein sequences were aligned and compared. Conserved amino acid residues are highlighted in red/yellow, weak similarities are highlighted in green, and gaps are marked with dashed lines (“–”). **(B)** Equal protein quantities from various C57BL/6 mouse (2-month-old) tissues were analyzed for SNX8 and GAPDH. SNX8 band intensities were quantified by densitometry, normalized to GAPDH; and compared to levels detected in heart (set to 1.0), *n* = 3. **(C)** Equal protein quantities from cultured primary neurons, astrocytes and microglia were analyzed for SNX8, NeuN (neuron marker), Iba1 (microglia marker), GFAP (astrocyte marker), and β-actin levels. SNX8 levels were quantified by densitometry, normalized to those of β-actin, and then normalized to levels detected in neurons (set to 1.0), *n* = 4. **(D)** HeLa cells co-transfected with SNX8-myc and EGFP-tagged Rab4, Rab5, or Rab7 constructs were immunostained for myc (to detect SNX8, red) and stained with DAPI (blue). HeLa cells transfected with SNX8-myc were immunostained for myc (SNX8, red) and Giantin (green), and stained with DAPI (blue). Images were acquired by confocal microscopy. Rab4, Rab5, Rab7, and Giantin staining is depicted in green. Zoom images are magnified panels from regions in merged images. Scale bars, 10 μm.

A high degree of protein sequence conservation is observed in human, mouse and rat SNX8 (Figure 2A). To characterize SNX8 expression in mouse, we measured SNX8 protein levels in various tissues from 2-month-old C57BL/6 mice by Western blotting (Figure 2B). SNX8 was ubiquitously expressed in all tissues examined, with relatively lower expression levels in brain. SNX8 expression was found to be slightly higher in microglia compared to neurons and astrocytes (Figure 2C). We examined SNX8 localization to various endosomal subcompartments by immunofluorescence, and observed that SNX8 predominantly localized to early endosomes as indicated by colocalization with Rab5. SNX8 also showed partial colocalization with early/recycling endosomes (Rab4), late endosomes (Rab7), and Golgi (Giantin) (Figure 2D). These results suggest that SNX8 is expressed to some extent in the brain, and predominantly localizes to endosomal compartments.

### SNX8 Modulates APP Levels, and sAPPα and Aβ Secretion

**FIGURE 3:**
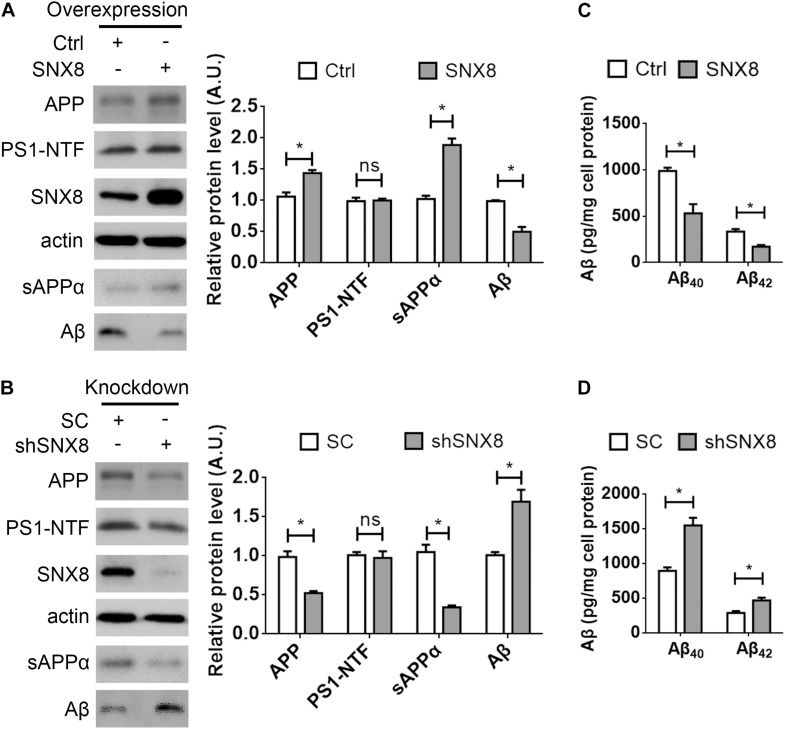
SNX8 Modulates APP, Aβ, and sAPPα Levels. **(A,B)** HEK-swAPP cells were transfected with control (Ctrl) and SNX8 constructs for 48 h **(A)**, or transfected with scrambled control (SC) and SNX8 shRNA vectors (shSNX8) for 72 h **(B)**. Cell lysates were subjected to Western blotting for APP, PS1-NTF, SNX8, and β-actin. Aβ and sAPPα in conditioned media were analyzed by Western blotting (sAPPα) or immunoprecipitation-Western blotting (Aβ). APP, Aβ, PS1-NTF, and sAPPα band intensities were quantified by densitometry, and normalized to respective controls (set to 1.0 arbitrary unit, A.U.). *n* = 4, ns: not significant, ^∗^*p* < 0.05, Mann–Whitney *U* test. **(C,D)** Aβ_40_ and Aβ_42_ levels in conditioned media from cells overexpressing SNX8 **(C)** and SNX8 knockdown **(D)** were determined by ELISA and compared to respective controls. *n* = 4, ^∗^*p* < 0.05, Mann–Whitney *U* test.

Our results so far indicate that SNX8 levels are altered in human AD and APP/PS1 AD mouse brain. Since APP trafficking from endosomes can specify consequent processing through amyloidogenic or non-amyloidogenic pathways, alterations in endosomal SNX8 could potentially impact Aβ generation through endosomal APP trafficking. To test this, we overexpressed SNX8 in HEK-swAPP cells and observed that elevations in SNX8 resulted in a marked increase in total APP protein levels (Figure 3A). In addition, elevations in sAPPα were accompanied by reductions in Aβ in conditioned media with SNX8 overexpression (Figure 3A), suggesting that SNX8 may enhance non-amyloidogenic APP trafficking. In agreement with this notion, SNX8 depletion through the expression of SNX8-targeting shRNAs markedly reduced APP and sAPPα levels, and increased Aβ levels (Figure 3B). Either overexpression or knockdown of SNX8 had little effect on presenilin 1 amino-terminal fragment (PS1-NTF) levels (Figures 3A,B). Using ELISA assays to quantify Aβ levels in conditioned media, we confirmed that SNX8 overexpression reduced secreted Aβ_40_ and Aβ_42_ levels while SNX8 depletion by shRNA transfection increased Aβ_40_/Aβ_42_ levels in conditioned media (Figures 3C,D). Together, these results indicate a role for SNX8 in enhancing non-amyloidogenic APP processing.

### SNX8 Modulates APP Stability

**FIGURE 4:**
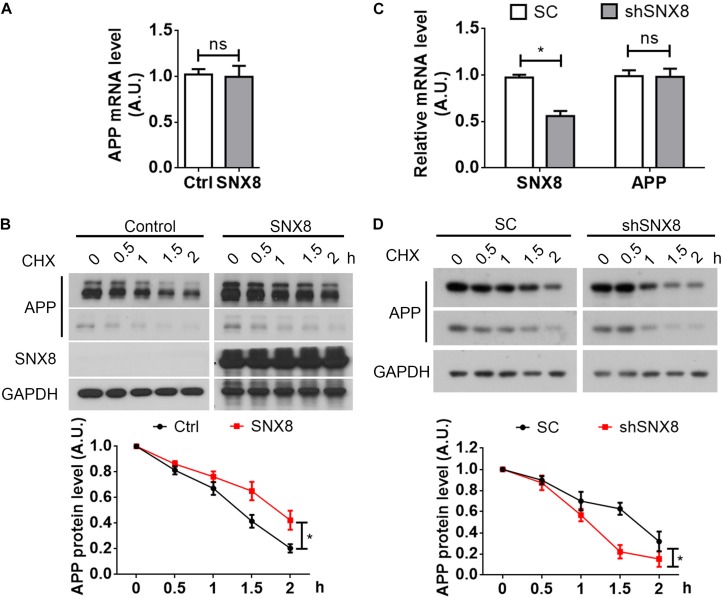
SNX8 Modulates APP Stability. **(A)** SH-SY5Y cells were transfected with SNX8-myc (SNX8) or control (Ctrl) vectors for 24 h. APP mRNA levels were determined by quantitative real-time PCR, normalized to β-actin levels, and compared to controls (set to 1.0), *n* = 4. ns: not significant, Mann–Whitney *U* test. **(B)** After transfection with SNX8-myc (SNX8) or control vectors for 24 h, SH-SY5Y cells were incubated with 500 μM cycloheximide (CHX) for the time indicated. Equal protein quantities from lysates were subjected to Western blotting. APP levels were quantified by densitometry, normalized to those at time point zero (set to 1.0), and compared to respective controls, *n* = 4, ^∗^*p* < 0.05, two-way ANOVA. **(C)** HEK293T cells were transfected with APP-myc for 24 h, then transfected with scrambled control (SC) or SNX8 shRNA (shSNX8) vectors for 48 h. SNX8 and APP mRNA levels were determined by quantitative real-time PCR, normalized to β-actin levels, and compared to respective controls (set to 1.0), *n* = 4, ^∗^*p* < 0.05, ns: not significant, Mann–Whitney *U* test. **(D)** After transfection with APP-myc for 24 h and scrambled control (SC) or SNX8 shRNA (shSNX8) vectors for 48 h, HEK293T cells were incubated with 500 μM CHX for the time indicated. Equal protein quantities from lysates were subjected to Western blotting. APP levels were quantified by densitometry, normalized to those at zero time point (set to 1.0), and compared to respective controls, *n* = 4, ^∗^*p* < 0.05, two-way ANOVA.

Trafficking of various protein components from the endosome can potentially influence their transport and subsequent degradation in lysosomes (Jiang et al., 2014). Given that SNX8 levels can affect total APP levels, we determined here whether SNX8 overexpression could affect APP stability. Although we observed little or no influence in APP mRNA levels with SNX8 overexpression (Figure 4A), we found that SNX8 overexpression significantly attenuated APP turnover in the presence of cycloheximide (Figure 4B). Consistently, knockdown of SNX8 had little effect on APP mRNA levels (Figure 4C), and significantly accelerated APP turnover in the presence of cycloheximide (Figure 4D). These results indicate that SNX8 can enhance APP stability with little or no influence in APP mRNA transcription.

### SNX8 Regulates APP Trafficking to the Cell Surface

**FIGURE 5:**
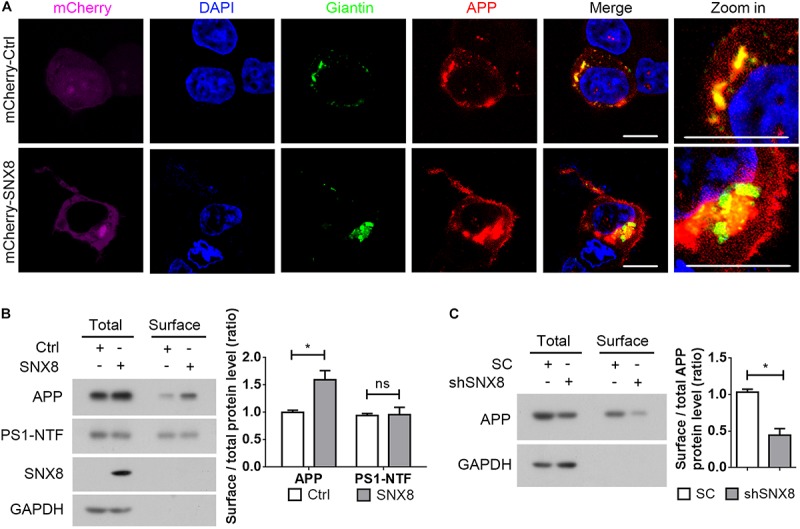
SNX8 Regulates APP Trafficking to the Cell Surface. **(A)** HEK293T cells were co-transfected with APP-myc and mCherry control (mCherry-Ctrl) or mCherry-SNX8 vectors for 24 h. Cells were permeabilized, fixed, immunostained with antibodies against myc (to detect APP, red-pseudo color) and Giantin (to detect Golgi, green-pseudo color), and stained with DAPI (blue). Images were acquired by fluorescence microscopy. mCherry was shown in magenta (pseudo color). Scale bars, 10 μm. **(B)** HEK293T cells were co-transfected with APP-myc and SNX8-myc (SNX8) or control (Ctrl) vectors for 24 h, and subjected to cell surface biotinylation. Biotinylated cell surface components were precipitated from cell lysates with streptavidin-agarose beads. Cell surface biotin-labeled APP, PS1-NTF, and inputs from the cell lysates were detected by Western blotting. Cell surface APP (or PS1-NTF) and total APP (or PS1-NTF) intensities were quantified by densitometry, and the ratio of cell surface APP (or PS1-NTF) levels to total APP (or PS1-NTF) levels was compared to the control ratio (set to 1.0). *n* = 4, ^∗^*p* < 0.05, ns: not significant, Mann–Whitney *U* test. **(C)** HEK293T cells were transfected with APP-myc and scrambled control (SC) or SNX8 shRNA (shSNX8) vectors for 48 h. After cell surface biotinylation, biotinylated proteins were precipitated for Western blotting. Cell surface APP and total APP intensities were quantified by densitometry, and the ratio of cell surface APP levels to total APP levels was compared to the control ratio (set to 1.0). *n* = 4, ^∗^*p* < 0.05, Mann–Whitney *U* test.

Since sAPPα generation through APP cleavage by α-secretases predominantly occurs at the cell surface (Parvathy et al., 1999), it is likely that elevations in sAPPα with SNX8 overexpression is derived from enhanced APP sorting to the cell surface. We noted that while APP was markedly localized to the Golgi (Hartmann et al., 1997; Xu et al., 1997; Greenfield et al., 1999), overexpression of SNX8 resulted in redistribution of APP from the Golgi compartments to other subcellular sites (Figure 5A). In agreement with our observations that SNX8 can enhance sAPPα generation, overexpression of SNX8 also enhanced the ratio of cell surface APP levels to total APP levels, indicating an increased cell surface distribution of APP (Figure 5B). While SNX8 overexpression did not affect cell surface PS1-NTF levels (Figure 5B). Consistently, knockdown of SNX8 reduced the ratio of cell surface APP levels to total APP levels (Figure 5C). These results indicate that SNX8 can enhance APP trafficking from intracellular compartments, and promote its redistribution to the cell surface.

### AAV-Mediated SNX8 Overexpression Ameliorates Aβ Accumulation and Cognitive Defects in APP/PS1 Mice

**FIGURE 6:**
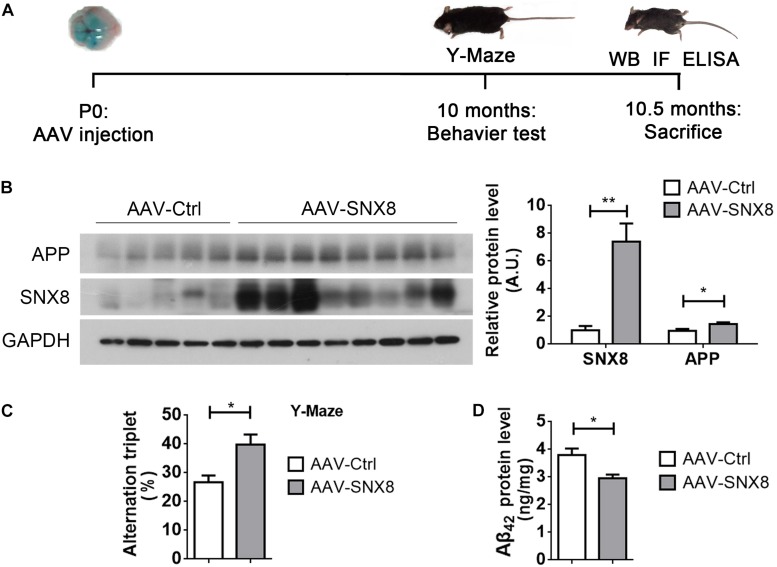
SNX8 Overexpression Improves Short-Term Memory and Reduces Aβ_42_ in APP/PS1 Mice. **(A)** A workflow diagram for AAV-SNX8 injection and subsequent analysis. **(B)** Equal protein amounts of hippocampal lysates from APP/PS1 mice injected with AAV-SNX8 or controls (AAV-Ctrl) were subjected to Western blotting for APP, SNX8, and GAPDH. APP and SNX8 band intensities were quantified by densitometry, and normalized to those of GAPDH for comparison (samples injected with AAV-Ctrl were set to 1.0 arbitrary unit, A.U.). AAV-SNX8, *n* = 8; AAV-Ctrl, *n* = 5. ^∗^*p* < 0.05, ^∗∗^*p* < 0.01, Mann–Whitney *U* test. **(C)** APP/PS1 mice injected with AAV-SNX8 and controls were evaluated for memory-related behavior in Y maze tests. Spontaneous alternations were determined between experimental groups. AAV-SNX8, *n* = 8; AAV-Ctrl, *n* = 5. ^∗^*p* < 0.05, Mann–Whitney *U* test. **(D)** Aβ_42_ levels in hippocampal lysates from injected mice were analyzed by ELISA. AAV-SNX8, *n* = 8; AAV-Ctrl, *n* = 5. ^∗^*p* < 0.05, Mann–Whitney *U* test.

So far, our results indicate that reductions in SNX8 levels in human AD and mouse APP/PS1 AD models may potentially enhance amyloidogenic APP processing to promote Aβ accumulation and cognitive impairment. This suggests that SNX8 overexpression may attenuate amyloidogenic APP processing and cognitive deficits. To test this, we injected AAV particles containing a SNX8 expression cassette (AAV-SNX8) into the bilateral brain ventricles of P0 APP/PS1 mice. Mice at 10 months of age were then subjected to behavioral analysis using Y-maze tests, whereby mice were sacrificed and brain tissue was processed for biochemical analysis (Figure 6A). We observed a marked elevation of SNX8 protein levels and an increase of total APP protein levels in hippocampi of mice subjected to microinjection with AAV-SNX8 compared to control AAVs (Figure 6B). Interestingly, we observed that explorative Y-maze alternation score of APP/PS1 animals injected with AAV-SNX8 was markedly improved compared those injected with AAV controls (Figure 6C), indicating that SNX8 overexpression can ameliorate behavioral alternation phenotypes in AD models. As our results above indicate that SNX8 overexpression can reduce Aβ levels in cell models, we similarly determined whether AAV-mediated SNX8 overexpression can attenuate Aβ_42_ levels in APP/PS1 mice. Indeed, we observed decreased Aβ_42_ levels in the hippocampus of AAV-SNX8 injected APP/PS1 mice compared to those of APP/PS1 controls (Figure 6D). Together, these results indicate that SNX8 overexpression can attenuate Aβ accumulation and reverse cognitive behavioral impairments in the APP/PS1 AD mouse model.

## Discussion

Single nucleotide polymorphisms within the *SNX8* gene locus have been previously associated with human AD (Rosenthal et al., 2012), however, it was unclear whether SNX8 is dysregulated in AD, and whether SNX8 may play a role in AD pathogenesis. Our results here indicate that SNX8 levels are attenuated in human AD and mouse APP/PS1 AD models; SNX8 mediates APP trafficking from amyloidogenic endosomal compartments, and suppresses Aβ generation and cognitive impairment in APP/PS1 mice. SNX8 depletion may promote APP trafficking to amyloidogenic endosomal compartments for increased Aβ production and accelerated APP cleavage and degradation. Together, these results strongly implicate a role for SNX8 in amyloidogenic APP trafficking and AD onset. It remains to be seen whether other SNXs are genetically associated with AD onset, and whether the expression and function of other SNXs are dysregulated in AD.

Changes in the endosome occur early during AD pathogenesis, where enlarged neuronal endosomes have been described in AD brain (Cataldo et al., 2000). Alterations in endocytosis manifest early in AD, where aberrant morphological changes in endosomes occurs decades prior to AD onset (Cataldo et al., 1997; Cataldo et al., 2000). This suggests that impairment of endocytic machinery can be an early pathogenic event driving neurological dysfunction in AD onset. Our observations that SNX8 levels are reduced early in APP/PS1 mouse models indicates that SNX8 depletion may play a key role in promoting amyloidogenic APP processing and enhancing Aβ generation. Given that prolonged SNX8 overexpression can ameliorate cognitive impairment at late stages in APP/PS1 mice, it seems likely that restoration of SNX8 function at early stages of AD onset may confer protective effects during advanced stages of disease onset. Future work may determine whether modulation of SNX8, and other related endosomal trafficking components can be used in future therapeutic Aβ-targeting strategies in AD.

## Data Availability

The datasets generated for this study are available on request to the corresponding author.

## Ethics Statement

Research involving human samples was reviewed and approved by the Medical Ethics Committee of School of Medicine, Xiamen University. Animal Subjects: The animal study was reviewed and approved by the Laboratory Animal Management and Ethics Committee of Xiamen University.

## Author Contributions

YX, MN, CJ, CZ, YT, ZS, and CW performed the experiments. HX and YZ reviewed the manuscript. HL and DC edited the manuscript. TH, Y-wZ, and XZ wrote the manuscript. All authors read and approved the final manuscript.

## Conflict of Interest Statement

The authors declare that the research was conducted in the absence of any commercial or financial relationships that could be construed as a potential conflict of interest.
